# Supplementary material for: Timely initiation of antenatal care and associated factors among pregnant women attending antenatal care in Southwest Ethiopia
Source: PLoS One. 2022 Aug 18;17(8):e0273152. doi: 10.1371/journal.pone.0273152 (PMC9387795; doi:10.1371/journal.pone.0273152)
Supplement: Attachment — Submitted filename: Response to Reviewers.docx [file pone.0273152.s003.docx]

**Response to Reviewers and Editor**

Firstly, we would like to thank editor and reviewers for your valuable and constructive comments and questions that are helpful for improving the quality of the manuscript. The responses for each point are done as follows.

**To Editor**

1. The manuscript is done based on the journal requirements
2. The questionnaire as a supporting information is separately attached
3. The data availability statement is described within the manuscript
4. Regarding providing repository information, we have changed our mind. We will provide the necessary information upon request by the journal.
5. Ethics statement out of the methods section is deleted
6. Regarding some minor occurrence of overlapping text with the following publication (<http://www.bioline.org.br/request?rh13063>), we checked it and by now the whole manuscript is revised to avoid any overlap.

**Response to Reviewers**

**Reviewer #1:** Thank you for giving me the opportunity to review this paper.

My comments are the following:

**Background**

- The background should be written at a higher level as we can assume that readers have basic understanding of the topic. For example, ANC does not need to be defined.

**Response:** The whole background section is revised and necessary changes are made to make it at higher level and the definition of ANC is removed.

- The background should try to make a better link between timely initiation of ANC and maternal mortality

**Response**: A statement that describes the link between timely initiation of ANC and maternal mortality is added in the revised manuscript **(please see paragraphs 3 & 4** **of the current version)**

- I would suggest using local or regional references when discussing factors influencing timely initiation of ANC. The references are also quite outdated and would recommend using more current references such as:

https://bmcpregnancychildbirth.biomedcentral.com/articles/10.1186/s12884-020-03236-9

https://www.ncbi.nlm.nih.gov/pmc/articles/PMC7864666/

**Response:** Local and regional references are used to discuss the factors associated with timely initiation of ANC (Please see references 10-12) and all outdated references are replaced by the current references including the references you suggested us. **(Please see references 1-15)**

**Methods**

- The methods should be re-written for better flow

**Response:** Correction is made throughout the methods section to make the flow better.

- The section of population is very repetitive and can be summarized in one sentence

**Response:** Summarized in one sentence and the repetition is corrected **(Please see population section of the revised manuscript)**

- Is Agaro a rural, peri-urban or urban setting?

**Response:** Agaro is an urban setting. However, clients are coming from both the town and the surrounding rural areas to get ANC services.

- How did you choose the independent variables? Please include a brief discussion or some references.

**Response:** The description and the references are included (**Please see the variables section of the revised manuscript)**

- A section on operational definition is not needed and can be incorporated earlier in the methods section

**Response:** Deleted as per your suggestion.

- How many centres/ institutions were eligible? How did you choose the five centres? Did you expect to have differences between public and private institutions - is that why you selected a mix of both?

**Response:** In the town, there were two health centers, one governmental hospital, six private clinics and five health posts. Of these, five health facilities (three government health institutions (one hospital and two health centers) and two private clinics) that had well documented registration books and providing ANC services were eligible and included in the study. **(Please see study design, area and period section).** Yes, we expect differences between public and private institutions and mostly researchers are focusing on public facilities only. So, to ensure the external validity of the findings to similar settings both public and private facilities were included.

- The section on Ethical Considerations should be shortened

**Response:** Shortened as per your comment **(Please see Ethical Considerations section of the revised manuscript)**

**Results**

- Please re-visit the titles of the different sections of the results. I would suggest perhaps consolidating some of these.

**Response:** Revisit is done on the titles of the different section of the results. However, consolidating the titles was not made because each title discusses different issues and have its own table.

**Discussion**

- Please structure the discussion to include strengths, limitations and policy implications

**Response:** Strengths, limitations and policy implications are included **(Please see the last paragraph of the discussion section)**

In the discussion, the authors make mention several times that the study findings are in keeping with findings of studies in other places in Ethiopia. Could you elaborate on whether these other places are similar to your study setting?

**Response:** Some of the studies are conducted in Southern Ethiopia relatively nearest to the current study area. Some others are conducted in Northwest and Eastern Ethiopia. Overall, most of the areas are similar to the study setting. Some others are different. The health service provision culture in Ethiopia is more or less similar. However, there might be variation in terms of providing 1^st^ ANC visit and the factors associated to it from area to area within the country. That’s why the current study is conducted to appreciate that difference and design appropriate area specific interventions.

- Please review the paper for English language/ grammar.

**Response:** The whole manuscript is revised to correct grammatical and writing errors**.**

**Reviewer #2:** This descriptive study highlights an important issue in the unique setting of Town Agaro town in Ethiopia with potentials to be basis of actionable plans towards getting pregnant women to attend ANC in timely manner.

However, the issues that should be addressed are:

1. Impact of untimely ANC attendance was not well captured- maternal mortality was not linked to the issue under study; thus, and also the rationale for the study in SW Ethiopia was unclear.

**Response:** Impact of untimely initiation of ANC is discussed and linked to maternal mortality (please see paragraph 4 of the current version) and the rationale for the study is revised **(please see the last paragraph of the background section)**

2. The setting of study is not easily understood eg- a. what type of health institutions exist in Agaro from which the facilities used were drawn from? private with fee for service, primary, secondary, etc? it should be described in setting

**Response:** In the town, there were two health centers, one governmental hospital, six private clinics and five health posts. Of these, three government health institutions (one hospital and two health centers) and two private clinics that had well documented registration books and providing ANC services were eligible and included in the study. **(Please see study design, area and period section).**

b. How did the authors selected the ones used.

**Response:** All health facilities that had well documented registration books and were providing ANC services were eligible and included in the study. Then, the sample size was proportionally allocated to each eligible health facility as per their client flow.

c. What is meant by rural and urban areas in the same town as used as a variables

**Response:** Though the health facilities are found in the town, the clients were coming from both urban area (Agaro town) and rural areas surrounding the town to get ANC services. That is why, the variable place of residence was used.

d. source of data/figures- such as a. number of pregnant women in the town. b. the two-month report of 798 of which institution and whose data?

**Response:** The detail description is made **(Please see study design, area and period section).**

3.. Population- the term seriously sick as an exclusive criterion is subjective- does it mean mothers who were hospitalized?

**Response:** It doesn’t mean hospitalized mothers. What we excluded were pregnant women who came to the health facilities for ANC service but unable to make the interview due to their sickness.

3. Variables- Many Variables were not clearly defined-
a. is being a House wife synonymous with being economically unemployed?

**Response:** Being a house wife in our context is if a pregnant woman is engaged in raising children and taking care of her family and the family income is based on husband’s income. So, housewives are economically unemployed.

b. waiting time (where? -before seeing the health worker or collecting lab results?),

**Response:** Waiting time is deleted from the variables list since it is not discussed in the results section.

c. cost of services- (confusing as the background pointed to free services in Agaro health facilities).

**Response:** Since private health facilities were included in the study cost of service (paid for service) is included in the variables list.

d. The cash/money classification in ETB means little if not explained, standardized or used in a socioeconomic classification.

**Response:** It was done based on previous studies for sake of comparison. However, it was not found to be significant in the current study.

e. satisfaction with services- How can a future event determine the timeliness of ANC, in other words satisfaction can’t be assessed from the point at when they were yet to use the ANC services, they were yet to use the ANC services; except it meant satisfaction with previous use

**Response:** That was writing error. Corrected as ‘satisfaction with ANC services for previous pregnancy’.

4. Ethics- while the single pregnant under 18yrs could have parental permission, a married minor is not a legal minor she may give consent by herself.

**Response:** Thank you for the lesson you thought us. we removed that part. **(Please see Ethical Considerations section of the revised manuscript)**

5. Results- within the tables, variables should be stated not as questions

**Response:** All variables stated in question form are changed to statement form **(Please see tables 3 and 4)**

6. The use of language - the word Lottery, i guess meant balloting, 'intention of pregnancy' should be planned or unplanned pregnancy.

**Response:** The issue of lottery method is described in the current version of sample size and sampling procedures section. **(Please see sample size and sampling procedures section of the current version).** The variable ‘pregnancy planned’ is changed as ‘intention of pregnancy’ and the categories are changed as ‘planned pregnancy and unplanned pregnancy’. **(Please see table 4)**

7. Results should be discussed not re-stated in discussions. comparisons of trends or percentages with previous studies should be clear with possible reasons truly related to findings

**Response:** The problem of re-stating is corrected and we tried to clarify the possible reasons for comparisons of trends or percentages with the previous studies.

**Reviewer #3:**

**Abstract**

- Methods: Line 31-32: The authors should revise the statement "Structured and pretested face-to-face interview technique was used to collect data"

**Response:** Changed as “A structured and pre-tested face-to-face interviewer-administered questionnaire was used to collect data.”

- The authors should add the sampling method to the methods section.

**Response:** Added as “Systematic random sampling technique was employed to recruit pregnant women.”

**Background**

- Lines 58-59: WHO has provided an updated recommended number of ANC visits and the authors should replace it with what they have in their manuscript currently.

**Response:** Replaced **(please see paragraph 3 of the current version)**

- Lines 65-67: The sentences in these lines lack adequate citations.

**Response:** Citations are added **(please see paragraph 1 of the current version)**

- Line 68 and Line 72: its sub-Saharan Africa not Sub-Saharan Africa

**Response:** Corrected **(please see paragraphs 1 and 5 of the current version)**

**Methods**

- The authors should provide more detailed description of the study setting highlighting the health facilities and if possible, the interventions put in place to improve maternal health service utilization.

**Response:** The detail description of the study setting is made **(Please see study design, area and period section).**

**Sampling size and sampling procedure**

- The authors should provide the formula used to calculate the sample size and support it with the appropriate reference.

**Response:** The formula is provided with appropriate reference **(Please see reference 16)**

- The authors should categorically state the sampling method employed in the and clearly describe how it was used to recruit the pregnant women.

**Response:** Brief description is made in the current version of sample size and sampling procedures section.

**Data collection**

- How did the authors check for the reliability and validity of the questionnaire?

**Response:** We checked the reliability of the questionnaire using Cronbach’s alpha reliability test and the value was 0.83. The validity of the questionnaire was checked by conducting pre-test, training supervisors and data collectors and translating the questionnaire to the local language and translating back to English. **(Please see Data collection instruments and procedures section of the current version)**

**Analysis**

- Did the authors apply weighting in the analysis?

**Response:** We didn’t apply weighting in the analysis.

**Ethical consideration**

- The authors should provide the ethical clearance number.

**Response:** We put the ethical clearance number **(Please see Ethical Considerations section of the revised manuscript)**

**Results**

- Table 3 and Table 4: The authors should make sure that all the variables that look like questions have been revised to look like variables.

**Response:** All variables stated in question form are changed to statement form (Please see tables 3 and 4)

- Line 200 and 211: The headings in these lines should be changed to sentence case.

**Response:** The headings are changed to sentence case.

- Why did the authors not perform regression analysis for the variables in Table 1 and Table 2? Any special reason for not doing so?? I suggest the authors reanalyze the data to include those variables in the regression analysis.

**Response:** We did regression analysis for all the variables included in the study including those listed in Table 1 and Table 2. However, only four variables (including gravidity from table 2) in table 5 were found to be significantly associated with timely initiation of antenatal care.

**Reviewer #4:**

I appreciate the work of the authors on identifying the factors associated with timely initiation of antenatal care in southwest Ethiopia. The authors highlight a major public health concern with widespread implications in an understudied population. Conclusions are both valid and meaningful. However, I have highlighted below some significant analytical concerns that should be addressed in the paper:

MAJOR COMMENTS

The manuscript would benefit from heavy editorializing. The grammar can be strengthened to avoid detraction from the value of the study.

**Response:** The whole manuscript is revised to correct grammatical and writing errors.

**INTRODUCTION**

- I appreciate that the authors keep the introduction short and succinct.

**Response:** We tried to make it short and succinct by not missing the necessary information that should be included.

**METHODS**

- The description of the sampling method is a bit unclear. The authors write that the proportion of timely initiation of ANC is 35.4%. Was this number calculated from the present study, or from the cited 2014 manuscript? What is the rationale for assuming there is a 10% non-response rate? Could the authors please expand upon the lottery method mentioned in lines 122-123?

**Response:** We tried to describe the sampling method clearly. The proportion of timely initiation of ANC, 35.5% was taken from the cited 2014 paper to estimate the sample size of the current study. The rationale for taking 10% non-response rate was to compensate invalid filled questionnaires due to incompleteness and writing errors. The way the lottery method used to recruit the first pregnant women (starting point) is more briefed in the current version of the manuscript.

- The authors would benefit from further discussion of the format of the questionnaire used to collect data. Were questions open-ended, agree/disagree, yes/no, etc.?

**Response:** The questions included in the questionnaire were closed-ended **(Please see Data collection instruments and procedures section of the current version)**

**RESULTS**

- The authors write that 20% of women with prior pregnancies had an abortion. Were these all results of elective procedures, or does this figure include miscarriages?

**Response:** It includes both elective procedures and miscarriages based on the report we got from pregnant women through the interviewer-administered questionnaire.

- How did mothers know they were experiencing complications (UTIs/high blood pressure) before their first ANC visit? Did the first visit encourage them to seek subsequent care?

**Response:** It is talking about the complications for the previous pregnancy. When they attended their previous ANC, they knew their overall complication status.

**DISCUSSION**

- What is the rationale for comparing southwest Ethiopia to Nepal and Pakistan? The nations and cultures are entirely different, and although all three countries are low-to-middle income regions, it doesn't make sense to compare them directly in this context.

**Response:** Primarily, we tried to compare the study findings to the previous findings in other places of Ethiopia. To expand the comparison outside Ethiopia, we selected countries like Nepal, Pakistan and so on from developing countries. So, the rationale is expanding comparison of the findings to other developing countries assuming that they are similar in terms of health service utilization including ANC visit.

- Although it is logical, are there any sources that can support the claim that women who have other children feel more confident and therefore are less likely to seek timely care? If not, please mention that formal analyses have not confirmed the authors' suspicions.

**Response:** The explanation was based on evidence **(Please see reference 25).**

**CONCLUSION**

- The manuscript would benefit from a brief expansion of recommended public health practices.

**Response:** Modification is made

**Reviewer #5:** The introduction has set a good context for the study and methods are sound and clearly outline the procedures. This is a well written paper. However, a few comments were observed below.

**Introduction**

- There is a paper quoted here: ''WHO Geneva. Carla A, Tessa W, Blanc A, Van P, et al. ANC in developing countries, 352 promises, achievements, and missed opportunities; an analysis of trends, levels and 353 Differentials, 1990-2001. 2003''. This research from 2003 is quite old. It would be beneficial to more recent evidence to give us a better picture and trend of timing of ANC visits.

**Response:** Thank you really. All old references are removed and replaced by the recent ones (please see references 1-15 of the current version)

**Discussion**

- Expatiate on efforts to be made to increase pregnant women’s knowledge on ANC and the right time for commencement, to include some practical examples.

**Response:** Modification is done with some practical examples. **(Please see the last paragraph of the discussion section)**

- Why was the more recent EHDS from 2019 not used? The maternal health care chapter contains information ANC timing.

**Response:** We used EDHS 2016 report to cite the information ‘only 20% of women had their first ANC during the first trimester’. However, the mini-EDHS 2019 doesn’t have information regarding the percentage of women who had their first ANC during the first trimester. When the full 2019 EDHS is released, it might be included.
