# Supplementary material for: Timely initiation of antenatal care and associated factors among pregnant women attending antenatal care in Southwest Ethiopia
Source: PLoS One. 2022 Aug 18;17(8):e0273152. doi: 10.1371/journal.pone.0273152 (PMC9387795; doi:10.1371/journal.pone.0273152)
Supplement: Attachment — Submitted filename: Response to Reviewers.docx [file pone.0273152.s004.docx]

**Response to Reviewers and Editor**

Firstly, we would like to thank the academic editor and reviewers for your additional valuable and constructive comments and questions that are helpful for improving the quality of the manuscript. The responses for each point are done as follows.

**To Academic Editor**

- The points raised by reviewer #2 are addressed.
- A rebuttal letter, Revised Manuscript with Track Changes and Manuscript without track changes are prepared.

**Response to Reviewers**

**Reviewer #2:** This study has been conducted rigorously, with large enough sample size to represent the population of interest. The data supports the overall conclusion of the authors, however the major areas the authors need to look into is the language - words or sentences used caused difficulty in comprehension

1. the authors may be unwittingly passing the message that WHO has a 'recommended proportion of women' that should have timely ANC visit from the statements in lines 44, 77, 78- it should be re-worded.

**Response:** Thank you for your critical comment. Revision is done **(Please see the conclusion part of the abstract and the conclusion section in the main document)**

2. in the Result section

i. variables should not be stated as questions

**Response:** Corrected **(Please see table 2)**

ii. line 198- 'Times of ANC visit' rather - ' frequency of ANC visits'

**Response:** Corrected **(Please see Table 3)**

iii. Line 209 Does this complication made you start ANC rather -' ANC attendance due to complications' [should also not be in question format]

**Response:** Corrected **(Please see Table 4)**

3. in line 243-247- in discussing and comparing the proportion /percentage of women with timely ANC visit, the researchers repeatedly mention the phrase ' the finding was lower/ much higher' - it should be stated that it is percentage which is being discussed.

**Response:** Corrected **(Please see the second paragraph of the discussion section)**

4. In proffering possible underlying in discussion of findings - many were stating random reasons with little scientific or factual evidence or citations e.g.- line 248 possible reasons for differences between current finding and other studies, the authors gave a string of reasons - " awareness, service coverage commitment and training of health workers " but no explanation, discussion or citations

**Response:** Corrected **(Please see the second paragraph of the discussion section)**
