# Supplementary material for: Timely initiation of antenatal care and associated factors among pregnant women attending antenatal care in Southwest Ethiopia
Source: PLoS One. 2022 Aug 18;17(8):e0273152. doi: 10.1371/journal.pone.0273152 (PMC9387795; doi:10.1371/journal.pone.0273152)
Supplement: Attachment — Submitted filename: Response to Reviewers.docx [file pone.0273152.s005.docx]

**Response to Reviewers and Editor**

Firstly, we would like to thank the academic editor and reviewers for your additional valuable and constructive comments and questions that are helpful for improving the quality of the manuscript. The responses for each point are done as follows.

**To Academic Editor**

- The points raised by reviewer #2 such as clarifications in the reporting, methodology justification, and language/copyediting and the specific issues raised are addressed.
- A rebuttal letter, Revised Manuscript with Track Changes and Manuscript without track changes are prepared.

**Response to Reviewers**

**Reviewer #2:**

A. While the authors have attempted to address some of the comments , they failed to work on the general manuscript to improve its intelligibility in standard English as preiuosly advised - this is the major weaknes of this manuscript. They may do well to employ the services of an editor to help with the written language.

- **Response:** From cover page to last page revision is made to correct writing and grammatical errors as indicated in the tracked changed manuscript.

B. Explanations for findings are not satisfactory mostly differences were just generic, not much specific explanations of differences between thier findings and the cited works - eg in line 261-266 " The possible reasons for this difference might be due to the difference in accessing information regarding timely initiation of antenatal care visit, advocating the importance of timely initiation of antenatal care, and advising pregnant women on the early booking of ANC, educational status of the pregnant women (14-15, 26). In addition, methodological variations among studies and the difference socio-demographic characteristics of the study participants might be the possible reasons-

- **Response:** Big revision is made on the explanations **(Please see lines 267-280 of the track changed manuscript)**

C... specific isssues that should have been worked on and still noted are stated below

1. line 27- should be- late in pregnancy , not in their late pregnancy; check and reword line47; line 50-60

- **Response:** Changes are made as per the request. **(Please see lines 27, 48 and 59 of the track changed manuscript)**

2.line 87- the statement -"That is lower than the WHO standard (14, 15)' .-the statement s makes little sense and references dont match the statement (14 and 15)

- **Response:** Thank you. The statement doesn’t go with the sentences before and after it. So, it is deleted from the manuscript. References 14 and 15 match with the sentence after it and they are included there. **(Please see line 87 of the track changed manuscript)**

3.line 67- ensure - not assure

- **Response:** Corrected. **(Please see line 67 of the track changed manuscript)**

4.line 100- better desciption of health facilties and number is needed eg- state total numbr of all the goernment health facilities and priate seperatly, then state the numer used in the study

- **Response:** The description is corrected. **(Please see lines 101-106 of the track changed manuscript)**

5.line-121-'included in' not included to th study as written

- **Response:** Corrected. **(Please see line 124 of the track changed manuscript)**

6. line 118-121- repitatation of line 100- theat section is just for sample size determination

- **Response:** The repeated part is deleted. **(Please see lines 121-124 of the track changed manuscript)**

7. line 131- sampling interval (K = N/n) was 2- ...2 can only be for 1 halth facilty , not be same for different study sites with different proportionaly chosen sample sizes and total population

- **Response:** The sampling interval calculated for each health facility was the same. Since it was proportionally allocated, the sampling interval was the same. It was approximately 2 for each health facility. Modification is done to indicate that the sampling interval was the same for each health facility **(Please see line 134 of the track changed manuscript)**

8.line 132- should be ...By balloting not lottery

- **Response: Corrected** (Please see line 135 of the track changed manuscript)

9.line 197 - should be- fifty eight (20.4%) pregnant women have had abortions ..not ..."Of those pregnant women who have had pregnancy experience, 58 (20.4%) of them have experienced abortion."

- **Response:** Thank you. Corrected as suggested **(Please see lines 200-201 of the track changed manuscript)**

10.line 198--200- thirty six(12.9%)Women had experienced pregnancy related complications....,- not ...Women who have faced various pregnancy related complications (pregnancy induced hypertension, anemia, eclampsia, pre-eclampsia) that required hospitalization or additional treatment were 36 (12.9%).

- **Response:** Thank you. Corrected as suggested **(Please see lines 203-204 of the track changed manuscript)**

11.line 211- 212 - the women are not reporting , its the knowledge the study tested - so saying One hundred eighty (48%) pregnant women reported that four and above ANC visits are needed for pregnant women during normal pregnancy is not quite right--- rather say 48% had knowledge that (or knew that) pregnant women required four and above ANC visits

- **Response:** Thank you. Corrected as suggested **(Please see lines 217-218 of the track changed manuscript)**

12.line 219- 223- how can the women know that they had high Diastolic BP and UTI before ANC???? and started ANC for this cause? Do they have home visits from health workers that test and disgnose them at home before presenting?

- **Response:** This finding was obtained from self-report during the interview. Changes are made to indicate this. **(Please see lines 226-229 of the track changed manuscript)**

13.line 260-261- shoul be- 'The percentage was also higher compared to the finding of xxx% from a systematic review and meta-analysis of studies conducted in Ethiopia'.. not .....The percentage was also higher compared to a systematic review and meta-analysis study conducted in Ethiopia

- **Response:** Changes are made **(Please see lines 267-270 of the track changed manuscript)**

14. line 277-281- This might be because the more pregnant women know the time of first ANC visit, the more their timely initiation of ANC service will increase ...... this is not an explanation , the authors are stating/repeating the results in a different manner

- **Response:** Changes are made **(Please see lines 293-297 of the track changed manuscript)**
